# Cultivation and genomic characterization of novel methanogens from arid desert biocrust

**DOI:** 10.1093/ismeco/ycag013

**Published:** 2026-01-19

**Authors:** Weitao Tian, Eva Petrová, Sanae Sakai, Julius Eyiuche Nweze, Anne Daebeler, Roey Angel

**Affiliations:** Institute of Soil Biology and Biogeochemistry, Biology Centre CAS, České Budějovice, 370 05, Czechia; Faculty of Science, University of South Bohemia, 370 05, České Budějovice, Czechia; Institute of Soil Biology and Biogeochemistry, Biology Centre CAS, České Budějovice, 370 05, Czechia; Institute for Extra-cutting-edge Science and Technology Avant-garde Research (X-star), Japan Agency for Marine-Earth Science and Technology (JAMSTEC), Yokosuka, 237-0061, Japan; Institute of Soil Biology and Biogeochemistry, Biology Centre CAS, České Budějovice, 370 05, Czechia; Institut National de Recherche Scientifique, Centre Armand Frappier Santé et Biotechnologie, Laval, QC, Canada; Institute of Soil Biology and Biogeochemistry, Biology Centre CAS, České Budějovice, 370 05, Czechia; Institute of Soil Biology and Biogeochemistry, Biology Centre CAS, České Budějovice, 370 05, Czechia; Faculty of Science, University of South Bohemia, 370 05, České Budějovice, Czechia

**Keywords:** archaea, soil, ROS, desiccation, comparative genomics

## Abstract

Methanogens are strictly anaerobic archaea capable of energy conservation by methane production, yet their presence in oxic and arid environments challenges existing paradigms. In this study, we enriched and genomically characterized seven methanogenic cultures from desert biocrusts, affiliated with the genera *Methanobacterium*, *Methanosarcina*, and *Methanocella*. Six of these new enrichment cultures represent new species. Nonetheless, phylogenomic analyses revealed close genetic relationships with organisms from anoxic environments, indicating the absence of an evolutionary distinction. Comparative genomics exposed diverse though non-unique repertories of antioxidant (e.g. catalase, superoxide dismutase and desulfoferrodoxin), and desiccation-resistance genes (including genes for maintaining osmotic pressure and repair of cell wall and membrane), with *Methanobacterium* spp possessing the lowest gene abundance and diversity for oxygen and desiccation tolerance. Nevertheless, the occurrence of a Class I methanogen such as *Methanobacterium* in arid soils challenges the notion that members of this class are less oxygen tolerant than Class II. Pangenome analysis further uncovered unique genes enriched in membrane-associated functions and potentially non-functional stress-related genes. Via a global metagenomic survey we find that methanogens are underdetected in dryland soils, likely due to sequencing depth limitations. Our findings highlight previously overlooked methanogen diversity and ecological plasticity in oxic and desiccated habitats, and emphasize the need for further studies to elucidate their survival strategies.

## Introduction

Methanogens are a group of *Archaea* that reduce carbon dioxide (CO_2_), acetate (acetoclastic methanogenesis), or methyl compounds (methylotrophic methanogenesis) to produce methane (CH_4_) as final metabolite [1, 2]. Methane has gained increasing attention for its significant contribution to climate change, as it is a 84-fold more potent greenhouse gas than CO_2_ over a 20-year period [3]. Methanogens are the main biological source of CH_4_, and therefore play a crucial role in the global CH_4_ budget [4, 5]. They are strict anaerobes, and are commonly found in anoxic environments, such as sediments, rice paddies, and animal guts [6, 7]. Methanogenesis, which predates the appearance of O_2_ in the atmosphere [8, 9], represents a form of anaerobic respiration [1, 10]. Phylogenetic analysis based on the SSU rRNA gene and conserved ribosomal protein markers showed that methanogens are not monophyletic but comprise two distantly related groups, designated as Class I and Class II [11, 12]. Of the six recognized orders, Class I includes *Methanobacteriales*, *Methanococcales,* and *Methanopyrales*, and Class II, includes *Methanosarcinales*, *Methanomicrobiales,* and *Methanocellales* [13–15]. Additionally, recent discoveries have further expanded the known methanogen diversity, including *Methanomassiliicoccales* [16, 17], and novel lineages in the phyla *Korarchaeota* [18], *Thermoproteota* (*Methanomethylicia*) [19], and unique methanogenic lineages outside *Euryarchaeota* [20].

In recent years, several genera of methanogens have been detected in different environments that experience either a steady flux of oxygen, such as rice roots [15], wetlands [21], and lake water [22], or are mostly oxic, such as oxygenated sandy sediments, savannas, and even surface desert soils [23–27]. The genera *Methanobacterium*, *Methanosarcina* and *Methanocella* have consistently been found in above mentioned soils, including dryland areas [24, 28]. Notably, previous studies demonstrated that O_2_ tolerance in methanogens is strongly influenced by habitat, as strains form environments with fluctuating redox conditions show higher tolerance for O_2_ than strains from permanently anoxic habitats, even within the same genus [29–31]. Moreover, methanogens in aerated soils have been shown to become active and grow when oxygen levels drop, e.g. after rewetting of desiccated soil and sediment ecosystems, or when surrounding microbial community and soil texture modulating O_2_ penetration and creating micro-anoxic sites [22, 23, 28, 29, 32]. The presence and activity of methanogens in desert soils is striking, as it implies that rewetting events may create transient anoxic microenvironments facilitating methane production, highlighting an overlooked source of atmospheric methane. These upland soil and desert methanogens endure both oxygen fluctuations and the osmotic pressures of desiccation and rehydration. Accordingly, laboratory experiments have confirmed that *Methanobacterium*, *Methanosarcina*, and *Methanocella* can survive in oxidative environments for hours or even days [24, 28, 33, 34]. However, unlike some other strict anaerobes (e.g. *Clostridium* sp. and *Sporomusa* sp.) [35], methanogens do not form spores, and the mechanisms they use to cope with oxygen and desiccation remain largely unknown.

Several key cofactors required for methanogenesis are sensitive to the presence of oxygen (O_2_) and its derivatives, i.e. reactive oxygen species (ROS), including hydrogen peroxide (H_2_O_2_), hydroxyl radicals (-OH), and O_2_^−^ radicals [33, 36–38]. In all the methanogenic pathways, the pivotal final step of the reaction of coenzyme B with the methyl group bound to the coenzyme M (CH_3_-S-CoM) to produce CH_4_ and the heterodisulfide (CoB-S-S-CoM) is highly conserved in methanogens and the most O_2_-sensitive [39]. The [4Fe-4S]^2+^ clusters contained in key enzymes involved in the process (heterodisulfide – HDR) and the low-coordinated iron atoms bound to the substrate make this step especially susceptible to ROS damage [40, 41]. Metagenomic analyses have unveiled a range of mechanisms by which methanogens may cope with oxidative stress, including synthesing antioxidant enzymes that detoxify ROS, reducing ROS production, and repairing DNA and protein damage [42–44]. Antioxidant enzymes rely on reducing equivalents, usually supplied by small redox proteins (including rubredoxin and thioredoxin) [36, 45]. Moreover, methanogens, particularly *Methanosarcina barkeri*, possess functional antioxidant enzymes, including catalase and iron superoxide dismutase [46, 47], which could be upregulated under oxidative stress [48]. Both classes of methanogens encode for various antioxidant enzymes, but antioxidant-encoding genes are generally more abundant in Class II methanogens [49]. Additionally, methanogens possess repair enzymes such as MutS/MutL homologs [42], and methionine sulfoxide reductase [50], which protect against oxidative damage to nucleic acids and proteins, respectively. In response to desiccation stress, a variety of proteins in methanogens could play a protective role. Certain genera, including *Methanobacterium* and *Methanosarcina*, regulate osmotic pressure by accumulating compatible solutes, such as glycine betaine and proline, thereby maintaining cellular osmotic balance and stabilizing proteins [51, 52]. Moreover, *M. barkeri* has been shown to produce extracellular polymeric substances (EPS), which can help the cells retain more moisture and protect during desiccation [53]. *Methanosarcina* is also known to form biofilms that protect cells, with lipid synthesis upregulated to enhance membrane fluidity and aid in water retention [54]. Finally, homologues of late embryogenesis abundant (LEA) proteins, which are common in plants, have been identified in methanogens (e.g. *Methanocella paludicola*) [55, 56]. Such proteins may protect other proteins from being inactivated when partially dehydrated [55]. Despite these insights, most studies on methanogens in (transiently) oxic soils have focused on community-level changes in the environment, with limited molecular investigations regarding cellular mechanisms [28, 57]. Furthermore, all the studies using methanogen cultures outlined above were conducted using isolates from anoxic environments. This leaves a large gap in our understanding of the mechanisms responsible for ROS resistance in methanogens which exist and survive in oxic conditions.

In this study, we enriched and cultured new methanogens from desert biocrusts, sequenced their genomes, and performed comparative genomics with 35 genomes and metagenome assembled genomes (MAGs) from multiple closely related lineages. Our study aimed to identify the genetic traits that enable methanogens inhabiting upland soils to adapt to aerated and desiccated conditions.

## Materials and methods

### Enrichment and cultivation, methane measurement, and growth assessment

Biocrust samples (ca. top 3 mm) were collected from two adjacent Negev Desert sites in Israel: near the ancient Nabatean city of Avdat (30°47′N, 34°46′E), and near the Liman irrigation system (30°50′N, 34°45′E). The area is an elevated, hilly region (ca. 800 m.a.s.l.) with an average annual rainfall of 80–100 mm, classified as arid [58]. The local loess soil is typically dry and exposed to air for the majority of the year [59]. Previous studies have shown the presence and activity of members of *Methanosarcina* and *Methanocella* in the biocrust, but not in the underlying soil [24].

To enrich methanogens, 1 g of biocrust was added to 5 ml of sterile deionized water in two 25 ml Balch-type tubes sealed with black butyl stoppers (VWR), crimped with an Al-ring, and flushed with a N_2_/CO_2_ (80%:20% vol/vol; see Supplementary material for more details) gas mixture. Deionized water was used as a rehydration step to reactivate microbial cells [60]. Enrichment cultures were kept in the dark at 32°C (average *in situ* summer temperature) in a vinyl anaerobic chamber (Coy) without shaking. Same temperature was applied subsequently throughout *Methanobacterium* and *Methanosarcina* cultivation steps. Methane production was measured weekly using a gas chromatograph (Agilent GC 6850) equipped with a ShinCarbon ST Micropacked GC column (Restek) and a Flame Ionization Detector (FID, Agilent Technologies). After one month, 1 ml H_2_ (5% final conc.) and 1 ml of deoxygenated adapted Sekiguchi medium (pH 7.0, see Supplementary material) were added [61]. After another month, CH_4_ producing enrichments were transferred (1:20) into fresh medium with N_2_/CO_2_ (80%:20%) headspace. Two enrichment lines were set up: one for *Methanobacterium* with an addition of 5% H_2_ in the headspace, and another for *Methanosarcina* with Na-acetate (0.01 g l^−1^) in the medium. Hereafter, the following antibiotics was applied alternately: kanamycin (MP Biomedicals), ampicillin, penicillin G (Merck), and vancomycin (Carl Roth) each at 50 mg ml^−1^, 1 ml per l medium for each transfer, with the final concentration at 50 μg ml^−1^. Methane production was monitored regularly. The community composition was monitored every few months via molecular methods as described below. After 10 months, 1 ml of *Methanobacterium*-dominated enrichment (34.4% of total community, 90.6% of total methanogens, estimated using ddPCR, see below) was transferred to 100 ml DSMZ 1523 medium [62] in 250 ml Duran bottles, capped with sterile butyl rubber stoppers, with 50% H_2_ added to the headspace. A *Methanosarcina-*dominated (91% of total community) culture was established after 16 months, for which a similar transfer was set up into adapted DSM 960 medium ([63]; see Supplementary material) with N_2_/CO_2_ (80%:20%) in the headspace.

To specifically enrich *Methanocella* sp., ~20 g of biocrust was pre-incubated at room temperature in a custom-made soil microcosm [28] under moist (field water-holding capacity of 9%) and anoxic (N_2_:CO_2_, 80%:20%) conditions for four weeks to activate the anaerobic microbial community. Afterwards, 5 g of pre-treated soil was mixed with 40 ml of fresh water basal medium (pH 7.0) [64] in a sterile 100 ml Duran bottle anoxically. 10% H_2_ and 5% sterile air were added to the headspace after flushing with N_2_/CO_2_ (80%:20%) and the culture was incubated anoxically at 37°C. A 5% air exposure was empirically determined during preliminary tests, in which *Methanocella*-containing cultures tolerated transient O_2_ levels and showed quick resumed growth. The addition of air was designed to suppress the growth of other methanogens that are assumed to be less aerotolerant than *Methanocella*. We followed the optimal growth temperature of *M. paludicola* [15] to maximize *Methanocella* biomass in our enrichments. Methane production was monitored as stated above on a weekly basis. A mix of penicillin and kanamycin (final concentration at 50 μg ml^−1^ for each) was applied to all media to suppress bacterial growth. More details of media and enrichment lines are given in the Supplementary material 1. Images of the enriched methanogenic strains were obtained using a scanning electron microscope (Supplementary material 2).

### DNA extraction, sequencing, and genome assembly

Soil biocrust DNA was extracted with the FastDNA™ SPIN KIT for SOIL (MP Biomedicals), and culture DNA was extracted using a phenol-chloroform extraction method [65]. The proportion of methanogens to all prokaryotic community members in the cultures were determined through droplet digital PCR (ddPCR) on a Bio-Rad QX200 Droplet Digital PCR System using the following assays: universal prokaryotes (general 16S rRNA gene primers), universal methanogens metahnogens (*mcrA* primers), and specific methanogen genera (genus-specific 16S rRNA primers). Details of the primers used and the ddPCR assays are given in Table S1. The genus-specific ddPCR values were corrected to methanogen cell numbers by dividing by the number of 16S rRNA gene copies in the genomes of each target genus: 2 copies in *Methanobacterium* [66], 3 copies in *Methanosarcina* [67], and 2 copies in *Methanocella* [15].

When the cell numbers of *Methanobacterium*, *Methanosarcina*, and *Methanocella* reached of 1.9 × 10^6^, 2.1 × 10^6^, and 1.3 × 10^6^ per ml, respectively as determined by ddPCR, DNA was extracted from the enrichment cultures using the Quick DNA HMW Magbead Kit (Zymo Research) according to the manufacturer’s instruction after an overnight treatment of harvested cells in DNA/RNA shield solution (Zymo Research) at −20°C. Total DNA was sent to Novogene Co., Ltd. (Munich, Germany) for library preparation and HiFi long-read sequencing on a PacBio Revio platform. The FASTQ sequences were extracted from the BAM file format using the “bam2fastq” tool from the PacBio BAM toolkit [68]. The extracted reads were then de novo assembled with the “—meta” command of the metaFlye algorithm (v2.9.3; [69]). The assembly outputs were visualized with the Bandage software (v0.8.1; [70]), and the contigs were exported in FASTA format. Circular genomes underwent further quality evaluation using QUAST (v5.2.0; [71]) to assess the assembly metrics, BUSCO (v5.7.0; [72]) to evaluate genome completeness based on conserved single copy orthologs, and CheckM (v1.2.2; [73]) to assess the level of contamination and completeness. Method details for DNA extraction and sequencing on an Illumina platform of an additional set of four enrichment cultures are given in Supplementary material 3.

### Phylogenomics and comparative genomics

PacBio- and Illumina-derived genomes were classified using GTDB-Tk (v1.4) with the archaeal reference database [74, 75]. To provide context, 35 reference genomes and MAGs (completeness >80, contamination <5) from methanogens of the same respective families (i.e. *Methanobacteriaceae*, *Methanosarcinaceae*, and *Methanocellaceae*) were obtained from Genome Taxonomy Database. For each class, a set of 122 archaeal single-copy marker genes [75] was separately aligned, resulting in three alignment files [76–78] and trimmed using trimAl (v1.5; removed gaps >50%) [79]. Afterwards, three maximum-likelihood trees were generated with IQ-TREE (v2.1.1) applying the WAG evolutionary model and with 1000 bootstrap iterations [80]. One outgroup genome was included per tree (*M. mazei* for *Methanobacterium* and *Methanocella*, and *M. veterum* for *Methanosarcina*). The phylogenetic trees were visualized and annotated using iTOL [81]. Subsequently, the open reading frames (ORFs) of all genomes and MAGs were predicted using Prodigal [82]. A custom set of 60 HMM marker profiles (oxic and desiccation stress genes) was searched using “hmmsearch” [83] with an e-value cutoff of 1.0 × 10^−10^. The antioxidant gene set was selected according to Lyu & Lu [42] and Johnson & Hug [84]. These included genes involved in combating oxidative stress (peroxides removal, free radical removal, iron storage / redox buffer, Fe-S cluster assembly, DNA glycolyase / endonuclease, and S=O / S-S recovery), and desiccation related damage (osmotic pressure regulation and membrane and cell wall repair) in methanogens. A complete list of all 61 marker proteins is given in Table S2. Methods for ordination analysis and a global search in publicly available metagenomes are given in Supplementary material 4.

To allow species delineation, genome-wide Average Nucleotide Identity (ANI) and Average Amino Acid Identity (AAI) values were calculated between the seven newly enriched strains and selected genomes of closely related methanogens for *Methanobacterium*, *Methanosarcina*, and *Methanocella*, according to [85, 86] respectively. ANI and AAI heatmap matrices were visualized with ChiPlot [87].

### Pangenome construction and annotation

To further compare the genomic composition of methanogens obtained in this study with closely related ones, we conducted a pangenome analysis using Anvi’o (v8; [88]). Unique and shared genes were identified as described in Eren, 2025 ([89]; detailed in Supplementary material 5). Through the pangenome analysis we identified distinct genes in each of the newly sequenced genomes (hereafter referred to as unique genes). These gene lists were manually explored for the presence of genes related to stress tolerance. Selected genes were quantified across all genomes presented in this study and gene neighbourhoods of these stress-associated genes were visualized with the “Gene Graphic” online tool [90]. For further methods regarding protein analyses, please see Supplementary material 6.

## Results and discussion

### Cultivation and methanogenic activity

The occurrence of active methanogens in environments experiencing oxygen or periodic desiccation have been known for years [23, 24, 28, 34], but the genetic basis has not been systematically studied. In part, this is due to the lack of isolates and their genomes from such environments in public repositories. Here, we partially filled this gap by culturing and genomically characterising desert biocrust methanogens affiliated with genera systematically found in upland soils, namely with *Methanobacterium*, *Methanosarcina*, and *Methanocella*.

It is believed that cultures of methanogens require strictly anoxic conditions. These classical headspace conditions, i.e. under H_2_/N_2_/CO_2_, or N_2_/CO_2_, excluding O_2_, were applied in this study to successfully enrich *Methanobacterium* and *Methanosarcina*. However, members of the genus *Methanocella* are notoriously difficult to culture owing to their slow growth and reduced competitiveness with other H_2_-utilizing methanogens [15, 91]. Although O_2_ tolerance can vary depending on habitat characteristics, all *Methanocella* strains isolated to date were from rice paddy soils [15, 92, 93], where periotic expose to O_2_ through root-mediated transportation. Based on this fact which suggests that members of *Methanocella* genus may exhibit relatively higher O_2_ tolerance than many other methanogens [24], we regularly spiked the headspace of the *Methanocella* enrichment cultures with 5% lab air in order to favour them over the faster-growing *Methanosarcina* and *Methanobacterium* species.

The abundances of *Methanobacterium* spp, *Methanosarcina* spp, and *Methanocella* spp in the original soil crust samples were 7.0 × 10^4^, 6.5 × 10^2^, and 1.3 × 10^2^ cells per gram dry weight, representing 2.4 × 10^−4^%, 2.6 × 10^−6^%, and 5.2 × 10^−7^% of the total prokaryotic communities, calculated and normalized from ddPCR results. Although abundances are extremely low, similar findings have been reported by Hall *et al.*, who showed that aerotolerant methanogens present at low copy numbers in coastal sediments can nevertheless sustain ecologically significant methane emissions [23]. It suggests that rare methanogens, even when close to the detection limit, may still support critical ecosystem processes under favourable conditions [24, 32].

After eight, six, and two transfers of the *Methanobacterium*, *Methanosarcina*, and *Methanocella* cultures (i.e. on days 586, 503, and 129 of cultivation in each lines), respectively, a strong dominance of each of the target methanogens was achieved. The enrichment levels of the target methanogen genus, expressed as a percentage of total methanogens, were 99.98% for *Methanobacterium*, 99.96% for *Methanosarcina*, and 98.05% for *Methanocella*, and of the entire prokaryotic communities were: 22.4% for *Methanobacterium,* 91% for *Methanosarcina*, and 16.3% for *Methanocella*. Methane production rates during enrichment cultivation were (1.13 ± 1.10) × 10^−3^, (7.37 ± 7.75) × 10^−3^, and (1.05 ± 1.19) × 10^−3^ nmol d^−1^ cell^−1^ for *Methanobacterium*, *Methanosarcina*, and *Methanocella* enrichment cultures, respectively. The methane production observed in these biocrust enrichments were within the lower range of reported laboratory pure-culture rates when normalized per cell [15, 94, 95], but community and matrix effects (e.g. soil macromolecules fermentation) in enrichment cultures can alter per-cell activity. Therefore, direct comparisons with pure cultures are difficult to make at this point.

**Figure 1:**
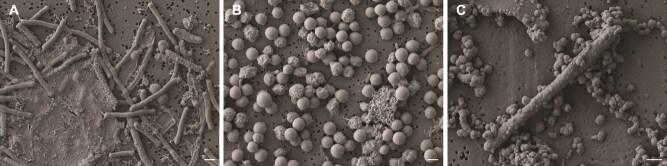
Scanning electron microscopy (SEM) images of the three newly enriched methanogens from desert biocrusts. *Methanobacterium* sp. (A) with a long rod shape, *Methanosarcina* sp. (B) with aggregated, coccoid cell clusters, and likely *Methanocella* sp. (C) with a shorter rod shape. The white line scale bars denote 1 μm.

The microscopic images obtained from scanning electron microscopy (SEM) analysis confirmed the typical cell (single-cell forms for *Methanosarcina*) morphologies and sizes for each methanogenic genus (Fig. 1).

Although the presence and even activity of methanogens in upland soils in general, and desert biocrust in particular, has been confirmed in the past [24, 28], the detection of Class I *Methanobacterium* spp is particularly striking. This is because Class I are thought to possess a smaller repertoire of antioxidant enzymes [42], and more [4Fe-4S] clusters [44, 96], making them more vulnerable to transient oxidative conditions than Class II methanogens, such as *Methanosarcina* and *Methanocella* [42, 44]. Additionally, unlike Class II, Class I methanogens do not possess cytochromes, which allow for energy conservation via membrane-bound electron transport chains and fuel protective mechanisms linked to increased oxygen resistance [96]. The successful enrichment and cultivation of methanogens including a cytochrome-lacking *Methanobacterium* from an environment which is mostly oxic points to the possibility of the existence of yet-undescribed oxygen survival and detoxification strategies in these organisms. These new enrichment cultures provide a valuable addition to the known methanogen cultures, which have all been obtained from anoxic habitats. They highlight a wider-than-expected ecological plasticity of methanogens and present a unique tool to further investigate potential oxygen tolerance and detoxification mechanisms.

### Genome characteristics and phylogeny

Using de novo assembly, we obtained seven distinct high-quality methanogen genomes from our enrichment cultures, four of which were circular: three classified as *Methanobacterium* spp. and three as *Methanosarcina* spp. (each two MAGs from the Illumina NovaSeq sequencing and one closed genome from PacBio sequencing), as well as one closed genome classified as belonging to a *Methanocella* sp. (closed genomes are shown in Fig. S1). Quality assessment using QUAST showed genome completeness of non-circular MAGs were 92.8, 93.6 (*Methanobacterium* spp.*)*, and 99.7, 99.8% (*Methano*sarcina spp.), with minimal contamination levels evaluated by CheckM at 0, 4 (*Methanobacterium* spp.), and 0.98, 0 (*Methano*sarcina spp.), respectively. Likewise, minimal contamination levels of closed genomes were determined with 0.8, 0.65, and 0% for the *Methanobacterium* sp., *Methanosarcina* sp., and *Methanocella* sp., respectively.

**Figure 2:**
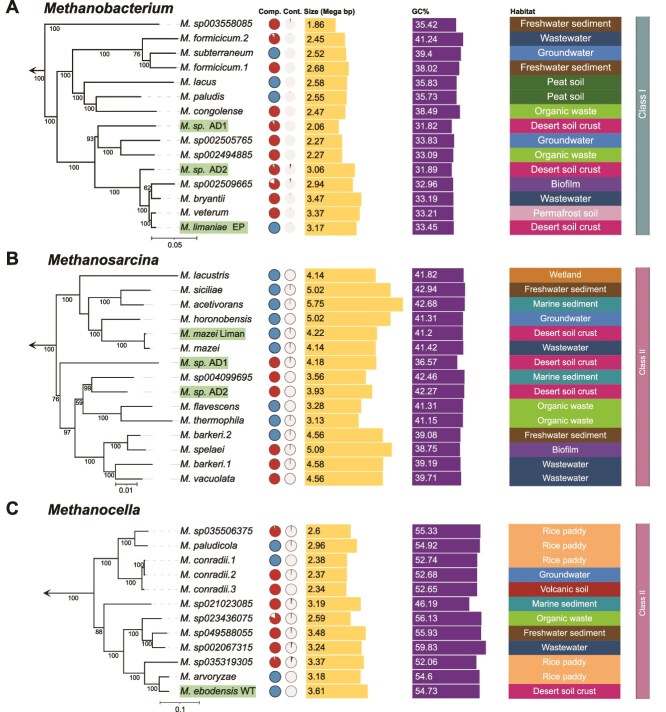
Phylogenomics and basic genome-assembly properties of the newly enriched methanogens and known representatives. Genome-based, maximum-likelihood trees for the genera *Methanobacterium* (A), *Methanosarcina* (B), and *Methanocella* (C) are shown with the newly enriched methanogens of this study highlighted in green. Numbers on branches indicate bootstrap support value (%) on 1000 replicates. The scale bars denote 0.05, 0.01, and 0.1 estimated substitutions per amino acid, respectively. The arrow at the base of each tree denotes rooting based on an outgroup (see Materials and methods for specifications). Comp., completeness (in red; blue solid circles represent circular genomes); Cont., contamination.

Phylogenomic analysis confirmed the affiliation of the organisms in our enrichment cultures with the *Methanobacteriales*, *Methanosarcinales*, and *Methanocellales* orders (Fig. 2, Table S3). Unexpectedly, the new methanogenic enriched cultures from a desert environment did not fall into separate clusters, but showed to be closely related to several known taxa from generally anoxic habitats such as wastewater, groundwater, permafrost, sub-sea sediments, or rice paddies. Moreover, we did not detect any phylogenomic clustering correlating with the environmental conditions in which the organisms live. The average genome sizes and G + C contents across the examined *Methanobacterium*, *Methanosarcina*, and *Methanocella* genera were 2.62 Mb and 35.9%, 4.39 Mb and 40.9%, 2.39 Mb and 50.4%, respectively. The variations in genome size and GC content among these methanogens may reflect their distinct metabolic strategies and environmental niches. Commonly, a lower GC content, such as found for the *Methanobacterium* spp., is associated with adaptation to anaerobic conditions [97, 98], which aligns with the classical view on Class I methanogens.

### Species delineation and distribution in global oxic drylands

**Figure 3:**
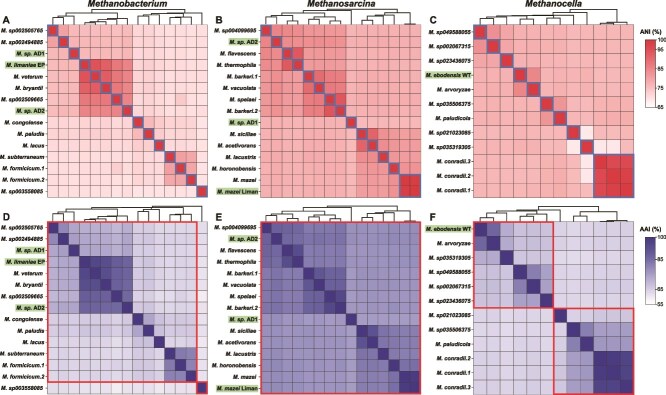
Genome-wide average nucleotide identity (gANI) and average amino acid identity (AAI) heatmaps for *Methanobacterium* (A, D), *Methanosarcina* (B, E), and for *Methanocella* (C, F). Names of genomes from this study are highlighted in green. Genomes within the same species in ANI heatmaps are framed in blue boxes, and genomes belonging to the same genera in AAI heatmaps are framed in red boxes.

Genome-wide average nucleotide identity (gANI) and average AAI based analyses were performed on the same *Methanobacterium*, *Methanosarcina*, and *Methanocella* datasets to classify the newly enriched desert methanogens on the species and genus levels and higher taxonomic ranks (Fig. 3). Based on the gANI analysis and a species level threshold of 96.5% [99] the three newly enriched *Methanobacterium* organisms represent separate species from each other and from *M. veterum* and *M. bryantii* (Fig. 3, Table S4) as their closest cultured relatives. After meeting the minimal standards proposed by Prakash *et al.* [100], for the species from which we obtained a closed genome, we therefore tentatively propose the name “*Candidatus* Methanobacterium limaniae EP”. We refrain from proposing new species names for the other two enrichment cultures, since they were only transiently present in the enrichment culture and moreover, we did not obtain circular genomes from them. Henceforth, we refer to these organisms as *Methanobacterium* sp. AD1 and AD2. Likewise, two of the newly enriched *Methanosarcina* organisms are separate species from each other and from *Methanosarcina flavescens* and *M. barkeri* 2, as their closest cultured relatives. We refer to these organisms as *Methanosarcina* sp. AD1 and AD2 from hereafter. The third *Methanosarcina* organism was not a separate species from *M. mazei* and we therefore give the strain name “Liman” to this organism. Finally, the newly enriched *Methanocella* organism was shown to represent a new species, distinct from its closest cultured relative *Methanocella aevoryzae*. We therefore tentatively propose [100] the name “*Candidatus* Methanocella ebodensis WT”. In summary, we have enriched and identified six novel methanogen species through genomic analysis of enriched organisms. The difficulty in cultivating and isolating methanogens, especially those with specialized metabolic requirements, has long been a challenge in microbial ecology. The identification of new species from three genera inhabiting oxic desert biocrusts in this study highlights the underappreciated diversity of methanogens in oxic habitats.

To assess the distribution of methanogens in arid oxic soils worldwide, we surveyed 24 metagenomic datasets collected from the NCBI Bioproject database generated from samples from Europe, the Americas, Asia, Africa, Oceania, and Antarctica. Methanogens were detected in only five of the 24 projects (44 datasets, Table S5 and S6). Among the detected methanogens, members of the class *Methanosarcinia* were most frequently observed. In addition, members of other methanogen classes including *Methanomicrobia*, *Methanococci*, *Methanobacteria*, and *Methanopyri* were all present in more than one dataset. However, the relative abundances were consistently low, representing max. 0.28% of the total microbial communities. Considering the discrepancy of apparent methanogen absence as assessed by metagenomic sequencing with the proven presence of methanogens via targeted approaches in this and previous work (e.g. for Negev desert biocrust [28]), we assume that the low detection rate of the global search reflects insufficient sequencing depth rather than their absence from these systems. Metagenomic surveys can underdetect low-abundance taxa due to limited sequencing depth, assembly and binning biases, however, low-abundance organisms may play disproportionate ecological roles [101, 102]. Therefore, targeted enrichment and deeper sequencing can help reveal their presence and potential function.

### Genomic potential for antioxidant and desiccation-resistance mechanisms in the newly enriched methanogens

**Figure 4:**
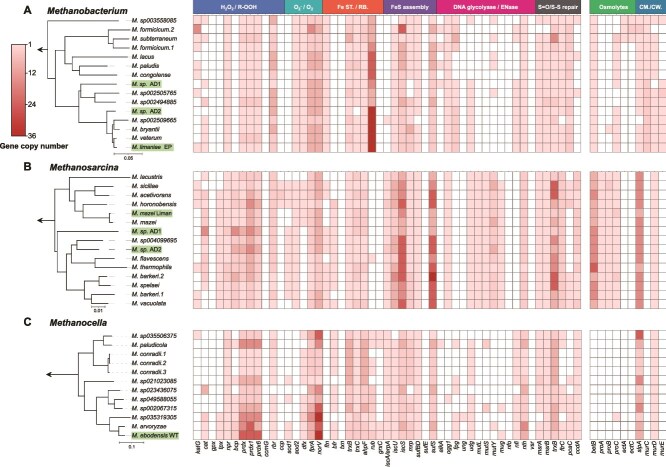
Distribution of distinctive genes related to antioxidant and desiccation stress traits in methanogens of the *Methanobacterium* (A), *Methanosarcina* (B), and *Methanocella* (C) genera. Genomes used in the analysis and phylogenomic trees on the left correspond to those in Fig. 2. A homology-based search for functional genes was performed by using HMMER’s hmmsearch and manual examination. Solid and open squares indicate the presence and absence of the genes, respectively. The presence of multiple copies is indicated by colour intensity. Gene names are given at the bottom (see Table S2 for full names), and the corresponding functions are annotated at the top. Fe ST., iron storage; RB., redox buffer; CM./CW., cell membrane/cell wall.

To identify genes associated with oxidative stress resistance and desiccation tolerance in the newly enriched methanogens, we conducted a targeted comparative genomic analysis across the genomes of our new enrichment cultures and those of their closely related relatives. There was a clear distinction in the encoded diversity of oxygen and desiccation stress genes between the three investigated species, indicating their different potential stress response strategies (Fig. 4, Fig. S2, Table S7). Notably, *Methanosarcina* genomes exhibited higher completeness in Fe-S cluster assembly and DNA glycosylase / endonuclease genes (Fig. 4B), which may confer an adaptive advantage to maintain genomic integrity even under harmful conditions that cause DNA lesions. Interestingly, the antioxidant gene set in *Methanocella* species was not the most complete among the three genera (Fig. 4C). This finding was unexpected, considering the generally attributed highest relative tolerance to oxygen of *Methanocella* and their ecological niche as rice-root methanogens [92, 103].

Surprisingly, the enriched methanogens from this study did not show a higher diversity, higher gene copy numbers, or specific patterns compared to their sister taxa within the genera. However, “*Ca.* Methanocella ebodensis WT” showed a modest increase in average copy number of antioxidant genes, ranking highest among the *Methanocella* genomes in this study. It aligns with our cultivation experiment, in which *Methanocella* demonstrated comparatively greater tolerance to O_2_ exposure than members of the *Methanobacterium*. The overall trends on the genus level remained apparent for our newly sequenced genomes with the three *Methanosarcina* genomes containing the highest diversity and copy numbers and the *Methanobacterium* genome containing the least (Fig. 4, Table S7). Previous studies showed that O_2_ tolerance can vary among strains belonging to the same genus depending on environmental exposure [29, 30]. Our findings likely reflect class and lineage level tendencies in antioxidant and desiccation-related gene repertoires. However, realized O_2_ tolerance is determined by gene regulatory differences, expression under stress, microhabitat protection, and community interactions, not only gene presence or absence. Future transcriptomic experiments and microcosm studies are required to resolve these layers.

The absence of distinctive genomic signatures uniquely associated with our desert strains suggests that these methanogens persistence under oxic and desiccating conditions may rely on mechanisms beyond gene content alone. One possibility is that the fine-structured matrix of biocrusts generates transient anoxic micro-niches, enabling methanogens to remain metabolically active during brief windows of O_2_ depletion [104]. Another explanation is regulatory plasticity, where antioxidant and repair pathways are rapidly upregulated in response to oxidative stress, even without an expanded gene repertoire. Additionally, physicochemical protection, such as encapsulation within soil aggregates, extracellular polymeric substances, or metabolic coupling with oxygen-scavenging community members, may help buffer methanogens from oxygen exposure [105–107]. Strains from habitats experiencing periodic oxygenation, even if phylogenetically close to strictly anaerobic strains, can have elevated tolerance for O_2_ [29, 30]. Such hypotheses requires transcriptomic and proteomic analysis, however, obtaining sufficient biomass for *Methanocella* sp. in particular remains extremely challenging. Future work combining controlled oxidative stress transcriptomic, targeted proteomics, and *in situ* microscale O_2_ profiling will be essential for resolving how methanogens tolerate and recover from oxygen and desiccation stress in desert.

Additionally, we specifically investigated the presence of LEA protein homologs (late embryogenesis abundant proteins) that were previously reported in *M. paludicola* genome and annotated as such by Campos *et al.* [55]. In plants, LEA proteins protect against dehydration stress by stabilizing proteins and membranes [108, 109], and have been discussed to provide similar functions in methanogens. However, our search using multiple archaeal LEA protein sequences from UniProt found zero hits across all *Methanocella* genomes including *M. paludicola*. A BLASTx search on the NCBI server showed that these *M. paludicola* LEA proteins annotated by Campos *et al.* have a high sequence similarity with KGG domain-containing proteins (Pfam domain PF10685) and general stress proteins rather than with LEA proteins (Table S8A). However, we did not find stress-induced KGG domain in those identified as “KGG domain-containing protein” based on domain analysis. To further examine this discrepancy, we compiled 19 LEA protein sequences from plants, bacteria, archaea, the five *Methanocella* putative LEA proteins, *et al.* bacterial KGG domain-containing proteins (uncharacterized protein YciG and Putative virulence-regulating protein), and an outgroup protein (hypersensitive response domain-containing protein), and constructed a neighbor-joining phylogenetic tree (Fig. S3A). In this tree, the five putative LEA proteins from *Methanocella* formed in a single separate cluster, sister to a plant LEA protein cluster. Sequence alignment likewise showed low similarity between these five proteins and canonical LEA proteins, as well as between them and KGG domain-containing proteins (Fig. S3B). Additionally, all five putative LEA proteins are highly hydrophilic and intrinsically disordered, which aligned with features of plant LEA proteins but not with archaeal LEAs. Finally, AlphaFold2 predicted the five *M. paludicola* proteins form extended, unstructured polypeptides with intermittent coiled α-helical segments, a feature of plant LEA proteins under stress-induced conformational changes [110, 111]. However, unlike archaeal LEA proteins exhibiting stable tertiary folds, the *M. paludicola* putative proteins lack any defined or persistent three-dimensional structures (Fig. S3C). Despite the physicochemical similarity with plant LEA proteins, these results raise doubts that *Methanocella* sp. truly harbour LEA proteins. We therefore caution against annotation of these sequences as LEA proteins or KGG domain-containing proteins without functional validation.

### Pangenomic analysis and potentially non-functional genes

To further explore genomic differences between the newly enriched desert methanogens and their close relatives, we conducted a pangenome analysis. The analysis identified both core genes shared across all genomes and unique genes specific to single genomes. All seven genomes of our newly cultivated methanogens contained unique genes, with the genome of “*Ca.* Methanocella ebodensis WT” containing the largest set (Fig. S4, Table S9). Strikingly, many (16%) of these unique genes in all our genomes were associated with membrane and cell wall biogenesis. In comparison, such genes accounted for 15% in *Methanobacterium*, 7% in *Methanosarcina*, and 10% in *Methanocella* among published genomes included in this study. The relative enrichment of such genes in our desert-derived strains may indicate lineage-specific strategies for maintaining cell integrity in the multi-factor extreme environment.

Additionally, several functionally intriguing genes were found, including those annotated as encoding subunits of glycosyltransferase (*rfaB*, *wcaA*, and *wcaE*) and the nitrous oxide reductase subunit D (*nosD)*. Glycosyltransferases are typically involved in exopolysaccharide biosynthesis and cell envelope modification, processes that may support stress resistance in bacteria [112, 113], but are poorly characterized in methanogens. The, *nosD* genes encodes for an accessory protein associated with the Nos operon for N_2_O reduction in denitrifiers [114], yet methanogens are not known to perform denitrification. To assess the distribution of these four genes, we quantified their copy numbers across all selected genomes in this study based on the anvi’o annotation (Fig. S5). The glycosyltransferase genes were widespread without genus-specific patterns. In contrast, *nosD* was enriched in *Methanosarcina* genomes only, implying a genus-specific functional adaptation possibly related to protein maturation. However, the putative *nosD* genes were not enriched in the genomes of the new desert methanogens, pointing towards a more general role.

To further understand the potential function of the gene annotated as *nosD* in our newly enriched methanogens, we performed a BLAST search against the NCBI nr protein database and out of the seven genes annotated as NosD, only three gave hits designated as NosD, albeit unverified (Table S8B). A Neighbor-joining phylogenetic tree built with *bona fide* NosD sequences showed that our putative NosD protein sequences formed a distinct cluster, separate from the other known ones (Fig. S6A). Additionally, synteny analysis of the putative *nosD* genes showed that other *nos* operon genes were absent (Fig. S7), suggesting that these genes may have alternative, yet unknown functions. Domain analysis showed that in addition to a distant periplasmic copper-biding (NosD-like) β-helix domain, these putative NosD proteins included pectin lyase folds, carbohydrate-binding / sugar-hydrolysis domains, and multiple parallel β-helix domains. Although the putative NosD proteins share similar domains with canonical ones, they were not classified within the representative “nitrous oxide reductase family maturation protein” by InterPro/PFAM databases (Fig. S6B). While the precise functions remain to be determined, these putative NosD proteins showed potential roles in cell-envelope modification, extracellular polymer processing, or general stress response, rather than denitrification [115–117].

In summary, although certain genes with intriguing annotations such as *nosD* and glycosyltransferases were found among the unique genes, their unclear functional roles, lack of operon context, and phylogenetic divergence suggest that they may represent non-functional remnants or genes with alternative, yet-to-be-elucidated roles in methanogens.

## Conclusion

The occurrence of methanogens of the *Methanbacterium*, *Methanosarcina*, and *Methanocella* genera in the investigated biocrusts defied basic assumptions about their physiology and left open questions regarding the restricted occurrence of only these genera and their genomic machinery to persist under the stressful conditions of these habitats. Our characterisation of newly-enriched methanogens showed that while they are equipped, to some extent, to handle ROS and desiccation, their genomes fail to reveal any unique mechanisms that would set them apart from their relatives living in anoxic and wet environments. Moreover, we found that *Methanocella*, which is regarded as the most oxygen tolerant methanogen, is in fact depleted in many known antioxidant genes compared to its sister genera. Concomitantly, we found that *Methanobacterium*, which was predicted to be particularly oxygen sensitive, is not only prevalent in oxic soils but also well-equipped to handle oxygen and desiccation stressors. Given the lack of strong genomic signals in the newly enriched methanogens from desert biocrust, it is possible that differences in gene expression contribute to their apparent stress tolerance. Therefore, in addition to ongoing cultivation and genomic sequencing attempts, further work on gene expression under physiologically-relevant stress conditions is required to elucidate the survival mechanisms of methanogens living in oxic and dry environments.

### Taxonomic consideration of “*Candidatus* Methanobacterium limaniae” sp. nov

Li.ma’ni.ae. N.L. gen. n. limaniae, belonging to the Liman, from Greek *λιμήν through Turkish and Russian, originally meaning a bay or a port, but in this context refers to a constructed runoff catchment, from which the strain has been isolated.*

Phylogenetically affiliated with the genus *Methanobacterium*, phylum *Methanobacteriota*. The genome consists of one circular chromosome of 3 168 381 bp. The DNA G + C content is 33.45 mol%. Strain “*Ca.* Methanobacterium limaniae EP” was cultivated from biocrust of the Negev desert, Israel. Soil hydrogenotrophic methanogen with a rod-shaped morphology. Cells are slender and elongated, typically measuring ~0.5 μm in diameter and 4–5 μm in length. The strain was routinely cultured with 80% H_2_ at 32°C in DSM17711 medium.

### Taxonomic consideration of “*Candidatus* Methanocella ebodensis” sp. nov

E.bo.den’sis. N.L. fem. adj. ebodensis, named after Eboda, a significant ancient Nabataean city situated along the “Incense Route,” close to the location where the strain was isolated. The Nabataeans were renowned for their sophisticated water collection techniques, which enabled them to thrive in the desert environment.

Phylogenetically affiliated with the genus *Methanocella*, phylum *Halobacteriota*. The genome consists of one circular chromosome of 3 614 771 bp. The DNA G + C content is 54.73 mol%. Strain “*Ca.* Methanocella ebodensis WT” was cultivated from biocrust of the Negev desert, Israel. Soil hydrogenotrophic methanogen with a rod-shaped morphology. Cells typically measure ~0.8 μm in diameter and 7–8 μm in length. The strain was routinely cultured with 10% H_2_ at 37°C in fresh water basal medium and subjected to 5% air regularly.

## Supplementary Material

Supplementary_material_revised_ycag013

Supp_table_revised_ycag013

## Data Availability

All sequencing data generated in this study have been deposited in the NCBI Sequence Read Archive (SRA) under BioProject accession number PRJNA1242298. Complete genome assemblies are available in NCBI GenBank under accession numbers CP187259.1, CP187260.1, and CP187261.1, and draft genome assemblies are available under accession numbers JBNAKP000000000.1, JBNAKN000000000.1, JBNAKM000000000.1, and JBNAKO000000000.1. The name of the novel methanogen strains “*Candidatus* Methanobacterium limaniae” sp. nov and “*Candidatus* Methanocella ebodensis” sp. nov have been validly registered under the SeqCode with the registry accession seqco.de/r:lhm19uk7 (https://seqco.de/r:lhm19uk7).

## References

[1] Blaut M . Metabolism of methanogens. *Antonie Van Leeuwenhoek* 1994;66:187–208. 10.1007/BF00871639 7747931

[2] Garcia PS, Gribaldo S, Borrel G. Diversity and evolution of methane-related pathways in archaea. *Ann Rev Microbiol* 2022;76:727–55. 10.1146/annurev-micro-041020-024935 35759872

[3] Masson-Delmotte V, Zhai P, Pirani AA. et al. (eds.). IPCC. Climate change 2021: The physical science basis. Contribution of Working Group I to the Sixth Assessment Report of the Intergovernmental Panel on Climate Change. Cambridge, United Kingdom and New York, NY, USA: Cambridge University Press, 2021.

[4] Kirschke S, Bousquet P, Ciais P. et al. Three decades of global methane sources and sinks. *Nat Geosci* 2013;6:813–23. 10.1038/ngeo1955

[5] Reeburgh WS . Oceanic methane biogeochemistry. *Chem Rev* 2007;107:486–513. 10.1021/cr050362v 17261072

[6] Boone DR, Whitman WB, Rouvière P. Diversity and taxonomy of methanogens. In: Ferry J.G. (ed.), Methanogenesis: Ecology, Physiology, Biochemistry & Genetics. Boston, MA: Springer US, 1993, 35–80.

[7] Garcia JL, Patel BKC, Ollivier B. Taxonomic, phylogenetic, and ecological diversity of methanogenic archaea. *Anaerobe* 2000;6:205–26. 10.1006/anae.2000.0345 16887666

[8] Battistuzzi FU, Feijao A, Hedges SB. A genomic timescale of prokaryote evolution: insights into the origin of methanogenesis, phototrophy, and the colonization of land. *BMC Evol Biol* 2004;4:44. 10.1186/1471-2148-4-44 15535883 PMC533871

[9] Ueno Y, Yamada K, Yoshida N. et al. Evidence from fluid inclusions for microbial methanogenesis in the early Archaean era. *Nature* 2006;440:516–9. 10.1038/nature04584 16554816

[10] Lyu Z, Shao N, Akinyemi T. et al. Methanogenesis. *Curr Biol* 2018;28:R727–32. 10.1016/j.cub.2018.05.021 29990451

[11] Bapteste É, Brochier C, Boucher Y. Higher-level classification of the archaea: evolution of methanogenesis and methanogens. *Archaea* 2005;1:353–63. 10.1155/2005/859728 15876569 PMC2685549

[12] Brochier-Armanet C, Forterre P, Gribaldo S. Phylogeny and evolution of the archaea: one hundred genomes later. *Curr Opin Microbiol* 2011;14:274–81. 10.1016/j.mib.2011.04.015 21632276

[13] Stadtman TC, Barker HA. Sudies on the methane fermentation x: a new formate-decomposing bacterium, *Methanococcus vannielii*. *J Bacteriol* 1951;62:269–80. 10.1128/jb.62.3.269-280.1951 14888644 PMC386125

[14] Kurr M, Huber R, König H. et al. *Methanopyrus kandleri*, gen. And sp. nov. represents a novel group of hyperthermophilic methanogens, growing at 110°C. *Arch Microbiol* 1991;156:239–47. 10.1007/BF00262992

[15] Sakai S, Imachi H, Hanada S. et al. *Methanocella paludicola* gen. Nov., sp. nov., a methane-producing archaeon, the first isolate of the lineage ‘Rice cluster I’, and proposal of the new archaeal order *Methanocellales* Ord. Nov. *Int J Syst Evol Microbiol* 2008;58:929–36. 10.1099/ijs.0.65571-0 18398197

[16] Iino T, Tamaki H, Tamazawa S. et al. *Candidatus* Methanogranum caenicola: a novel methanogen from the anaerobic digested sludge, and proposal of *Methanomassiliicoccaceae* fam. Nov. and *Methanomassiliicoccales* Ord. Nov., for a methanogenic lineage of the class *Thermoplasmata*. *Microbes Environ* 2013;28:244–50. 10.1264/jsme2.ME12189 23524372 PMC4070666

[17] Xie F, Zhao S, Zhan X. et al. Unraveling the phylogenomic diversity of *Methanomassiliicoccales* and implications for mitigating ruminant methane emissions. *Genome Biol* 2024;25:32. 10.1186/s13059-024-03167-0 38263062 PMC10804542

[18] Krukenberg V, Kohtz AJ, Jay ZJ. et al. Methyl-reducing methanogenesis by a thermophilic culture of *Korarchaeia*. *Nature* 2024;632:1131–6. 10.1038/s41586-024-07829-8 39048017

[19] Kohtz AJ, Petrosian N, Krukenberg V. et al. Cultivation and visualization of a methanogen of the phylum *Thermoproteota*. *Nature* 2024;632:1118–23. 10.1038/s41586-024-07631-6 39048824

[20] Wu K, Zhou L, Tahon G. et al. Isolation of a methyl-reducing methanogen outside the *Euryarchaeota*. *Nature* 2024;632:1124–30. 10.1038/s41586-024-07728-y 39048829

[21] Angle JC, Morin TH, Solden LM. et al. Methanogenesis in oxygenated soils is a substantial fraction of wetland methane emissions. *Nat Commun* 2017;8:1567. 10.1038/s41467-017-01753-4 29146959 PMC5691036

[22] Conrad R, Ji Y, Noll M. et al. Response of the methanogenic microbial communities in Amazonian oxbow lake sediments to desiccation stress. *Environ Microbiol* 2014;16:1682–94. 10.1111/1462-2920.12267 24118927

[23] Hall N, Wong WW, Lappan R. et al. Coastal methane emissions driven by aerotolerant methanogens using seaweed and seagrass metabolites. *Nat Geosci* 2025;18:854–61. 10.1038/s41561-025-01768-3 40949424 PMC12422968

[24] Angel R, Claus P, Conrad R. Conrad R methanogenic archaea are globally ubiquitous in aerated soils and become active under wet anoxic conditions. *ISME J* 2012;6:847–62. 10.1038/ismej.2011.141 22071343 PMC3309352

[25] Angel R, Pasternak Z, Soares MIM. et al. Active and total prokaryotic communities in dryland soils. *FEMS Microbiol Ecol* 2013;86:130–8. 10.1111/1574-6941.12155 23730745

[26] Angel R, Kammann C, Claus P. et al. Effect of long-term free-air CO_2_ enrichment on the diversity and activity of soil methanogens in a periodically waterlogged grassland. *Soil Biol Biochem* 2012;51:96–103. 10.1016/j.soilbio.2012.04.010

[27] Ma K, Lu Y. Regulation of microbial methane production and oxidation by intermittent drainage in rice field soil. *FEMS Microbiol Ecol* 2011;75:446–56. 10.1111/j.1574-6941.2010.01018.x 21198683

[28] Angel R, Matthies D, Conrad R. Activation of methanogenesis in arid biological soil crusts despite the presence of oxygen. *PLoS One* 2011;6:e20453. 10.1371/journal.pone.0020453 21655270 PMC3105065

[29] Wagner D, Pfeiffer EM, Bock E. Methane production in aerated marshland and model soils: effects of microflora and soil texture. *Soil Biol Biochem* 1999;31:999–1006. 10.1016/S0038-0717(99)00011-5

[30] Kiener A, Leisinger T. Oxygen sensitivity of methanogenic bacteria. *Syst Appl Microbiol* 1983;4:305–12. 10.1016/S0723-2020(83)80017-4 23194731

[31] Wagner D . Effect of varying soil water potentials on methanogenesis in aerated marshland soils. *Sci Rep* 2017;7:14706. 10.1038/s41598-017-14980-y 29089629 PMC5665900

[32] Conrad R . Methane production in soil environments—anaerobic biogeochemistry and microbial life between flooding and desiccation. *Microorganisms* 2020;8:881. 10.3390/microorganisms8060881 32545191 PMC7357154

[33] Fetzer S, Bak F, Conrad R. Sensitivity of methanogenic bacteria from paddy soil to oxygen and desiccation. *FEMS Microbiol Ecol* 1993;12:107–15. 10.1111/j.1574-6941.1993.tb00022.x

[34] Ueki A, Ono K, Tsuchiya A. et al. Survival of methanogens in air-dried paddy field soil and their heat tolerance. *Water Sci Technol* 1997;36:517–22. 10.2166/wst.1997.0631

[35] Madigan MT, Bender KS, Buckley DH. et al. Brock Biology of Microorganisms, Fifteenth edn. New York, Harlow (United Kingdom): Pearson ; Pearson Education limited, 2019.

[36] Imlay JA . Cellular Defenses against superoxide and hydrogen peroxide. *Annu Rev Biochem* 2008;77:755–76. 10.1146/annurev.biochem.77.061606.161055 18173371 PMC3057177

[37] Yuan Y, Conrad R, Lu Y. Transcriptional response of methanogen mcrA genes to oxygen exposure of rice field soil. *Environ Microbiol Rep* 2011;3:320–8. 10.1111/j.1758-2229.2010.00228.x 23761278

[38] Imlay JA . How oxygen damages microbes: oxygen tolerance and obligate anaerobiosis. *Adv Microb Physiol* 2002;46:111–53. 10.1016/s0065-2911(02)46003-1 12073652

[39] Kulkarni G, Kridelbaugh DM, Guss AM. et al. Hydrogen is a preferred intermediate in the energy-conserving electron transport chain of *Methanosarcina barkeri*. *Proc Natl Acad Sci USA* 2009;106:15915–20. 10.1073/pnas.0905914106 19805232 PMC2747218

[40] Duin EC, Madadi-Kahkesh S, Hedderich R. et al. Heterodisulfide reductase from *Methanothermobacter marburgensis* contains an active-site [4Fe–4S] cluster that is directly involved in mediating heterodisulfide reduction. *FEBS Lett* 2002;512:263–8. 10.1016/S0014-5793(02)02281-0 11852093

[41] Flint DH, Tuminello JF, Emptage MH. The inactivation of Fe-S cluster containing hydro-lyases by superoxide. *J Biol Chem* 1993;268:22369–76. 10.1016/S0021-9258(18)41538-4 8226748

[42] Lyu Z, Lu Y. Metabolic shift at the class level sheds light on adaptation of methanogens to oxidative environments. *ISME J* 2018;12:411–23. 10.1038/ismej.2017.173 29135970 PMC5776455

[43] Li J, Ran X, Zhou M. et al. Oxidative stress and antioxidant mechanisms of obligate anaerobes involved in biological waste treatment processes: a review. *Sci Total Environ* 2022;838:156454. 10.1016/j.scitotenv.2022.156454 35667421

[44] Lyu Z, Lu Y. Comparative genomics of three *Methanocellales* strains reveal novel taxonomic and metabolic features. *Environ Microbiol Rep* 2015;7:526–37. 10.1111/1758-2229.12283 25727385

[45] Lu J, Holmgren A. The thioredoxin antioxidant system. *Free Radic Biol Med* 2014;66:75–87. 10.1016/j.freeradbiomed.2013.07.036 23899494

[46] Shima S, Netrusov A, Sordel M. et al. Purification, characterization, and primary structure of a monofunctional catalase from *Methanosarcina barkeri*. *Arch Microbiol* 1999;171:317–23. 10.1007/s002030050716 10382262

[47] Brioukhanov A, Netrusov A, Sordel M. et al. Protection of *Methanosarcina barkeri* against oxidative stress: identification and characterization of an iron superoxide dismutase. *Arch Microbiol* 2000;174:213–6. 10.1007/s002030000180 11041352

[48] Brioukhanov AL, Netrusov AI, Eggen RIL. The catalase and superoxide dismutase genes are transcriptionally up-regulated upon oxidative stress in the strictly anaerobic archaeon *Methanosarcina barkeri*. *Microbiology* 2006;152:1671–7. 10.1099/mic.0.28542-0 16735730

[49] Erkel C, Kube M, Reinhardt R. et al. Genome of rice cluster I archaea the key methane producers in the rice rhizosphere. *Science* 2006;313:370–2. 10.1126/science.1127062 16857943

[50] Weissbach H, Resnick L, Brot N. Methionine sulfoxide reductases: history and cellular role in protecting against oxidative damage. *BBA - Proteins and Proteomics* 2005;1703:203–12. 10.1016/j.bbapap.2004.10.004 15680228

[51] Empadinhas N, Viete-Vallejo O. Osmoadaptation mechanisms in prokaryotes: distribution of compatible solutes. *Int Microbiol* 2008;**3**:151–61. 10.2436/20.1501.01.55 18843593

[52] Kempf B, Bremer E. Uptake and synthesis of compatible solutes as microbial stress responses to high-osmolality environments. *Arch Microbiol* 1998;170:319–30. 10.1007/s002030050649 9818351

[53] Anderson KL, Apolinario EE, Sowers KR. Desiccation as a long-term survival mechanism for the archaeon *Methanosarcina barkeri*. *Appl Environ Microbiol* 2012;78:1473–9. 10.1128/AEM.06964-11 22194299 PMC3294484

[54] Valentine DL . Adaptations to energy stress dictate the ecology and evolution of the archaea. *Nat Rev Microbiol* 2007;5:316–23. 10.1038/nrmicro1619 17334387

[55] Campos F, Cuevas-Velazquez C, Fares MA. et al. Group 1 LEA proteins, an ancestral plant protein group, are also present in other eukaryotes, and in the archeae and bacteria domains. *Mol Gen Genomics* 2013;288:503–17. 10.1007/s00438-013-0768-2 23861025

[56] Espelund M, Sæbøe-Larssen S, Hughes DW. et al. Late embryogenesis-abundant genes encoding proteins with different numbers of hydrophilic repeats are regulated differentially by abscisic acid and osmotic stress. *Plant J* 1992;2:241–52. 10.1046/j.1365-313X.1992.t01-46-00999.x 1302052

[57] Peng J, Lü Z, Rui J. et al. Dynamics of the methanogenic archaeal community during plant residue decomposition in an anoxic rice field soil. *Appl Environ Microbiol* 2008;74:2894–901. 10.1128/AEM.00070-08 18344350 PMC2394899

[58] Angel R, Conrad R. *in situ* measurement of methane fluxes and analysis of transcribed particulate methane monooxygenase in desert soils. *Environ Microbiol* 2009;11:2598–610. 10.1111/j.1462-2920.2009.01984.x 19601957

[59] Bruins HJ . Ancient desert agriculture in the Negev and climate-zone boundary changes during average, wet and drought years. *J Arid Environ* 2012;86:28–42. 10.1016/j.jaridenv.2012.01.015

[60] Placella SA, Brodie EL, Firestone MK. Rainfall-induced carbon dioxide pulses result from sequential resuscitation of phylogenetically clustered microbial groups. *Proc Natl Acad Sci USA* 2012;109:10931–6. 10.1073/pnas.1204306109 22715291 PMC3390866

[61] Sekiguchi Y, Kamagata Y, Nakamura K. et al. *Syntrophothermus lipocalidus* gen. Nov., sp. nov., a novel thermophilic, syntrophic, fatty-acid-oxidizing anaerobe which utilizes isobutyrate. *Int J Syst Evol Microbiol* 2000;50 Pt 2:771–9. 10.1099/00207713-50-2-771 10758888

[62] Leibniz Institute DSMZ-German Collection of Microorganisms and Cell Cultures . *1523: Modified Methanobacterium medium.* https://www.dsmz.de/microorganisms/medium/pdf/DSMZ_Medium1523.pdf. (22 April 2025, date last accessed).

[63] Leibniz Institute DSMZ-German Collection of Microorganisms and Cell Cultures . *960: Pelotomaculum medium.* https://www.dsmz.de/microorganisms/medium/pdf/DSMZ_Medium960.pdf. (14 May 2025, date last accessed).

[64] Tanaka S . Age estimation of freshwater sawfish and sharks in northern Australia and Papua New Guinea. *Nat Cult* 1991;3:71–82.

[65] Angel R, Petrova E, Lara-Rodriguez A. Total Nucleic Acids Extraction from Soil. *protocols.io*. https://www.protocols.io/view/total-nucleic-acids-extraction-from-soil-bwxcpfiw. (27 July 2021, date last accessed).

[66] Kelly WJ, Leahy SC, Li D. et al. The complete genome sequence of the rumen methanogen *Methanobacterium formicicum* BRM9. *Stand Genomic Sci* 2014;9:15. 10.1186/1944-3277-9-15 25780506 PMC4335013

[67] Maeder DL, Anderson I, Brettin TS. et al. The *Methanosarcina barkeri* genome: comparative analysis with *Methanosarcina acetivorans* and *Methanosarcina mazei* reveals extensive rearrangement within methanosarcinal genomes. *J Bacteriol* 2006;188:7922–31. 10.1128/jb.00810-06 16980466 PMC1636319

[68] PacificBiosciences/pbbioconda . 2024. PacBio, 2024.

[69] Kolmogorov M, Bickhart DM, Behsaz B. et al. metaFlye: scalable long-read metagenome assembly using repeat graphs. *Nat Methods* 2020;17:1103–10. 10.1038/s41592-020-00971-x 33020656 PMC10699202

[70] Wick RR, Schultz MB, Zobel J. et al. Bandage: interactive visualization of de novo genome assemblies. *Bioinformatics* 2015;31:3350–2. 10.1093/bioinformatics/btv383 26099265 PMC4595904

[71] Gurevich A, Saveliev V, Vyahhi N. et al. QUAST: quality assessment tool for genome assemblies. *Bioinformatics* 2013;29:1072–5. 10.1093/bioinformatics/btt086 23422339 PMC3624806

[72] Simão FA, Waterhouse RM, Ioannidis P. et al. BUSCO: assessing genome assembly and annotation completeness with single-copy orthologs. *Bioinformatics* 2015;31:3210–2. 10.1093/bioinformatics/btv351 26059717

[73] Parks DH, Imelfort M, Skennerton CT. et al. CheckM: assessing the quality of microbial genomes recovered from isolates, single cells, and metagenomes. *Genome Res* 2015;25:1043–55. 10.1101/gr.186072.114 25977477 PMC4484387

[74] Parks DH, Chuvochina M, Waite DW. et al. A standardized bacterial taxonomy based on genome phylogeny substantially revises the tree of life. *Nat Biotechnol* 2018;36:996–1004. 10.1038/nbt.4229 30148503

[75] Parks DH, Chuvochina M, Chaumeil PA. et al. A complete domain-to-species taxonomy for bacteria and archaea. *Nat Biotechnol* 2020;38:1079–86. 10.1038/s41587-020-0501-8 32341564

[76] Finn RD, Clements J, Eddy SR. HMMER web server: interactive sequence similarity searching. *Nucleic Acids Res* 2011;39:W29–37. 10.1093/nar/gkr367 21593126 PMC3125773

[77] Haft DH, Selengut JD, White O. The TIGRFAMs database of protein families. *Nucleic Acids Res* 2003;31:371–3. 10.1093/nar/gkg128 12520025 PMC165575

[78] Mistry J, Chuguransky S, Williams L. et al. Pfam: the protein families database in 2021. *Nucleic Acids Res* 2021;49:D412–9. 10.1093/nar/gkaa913 33125078 PMC7779014

[79] Capella-Gutiérrez S, Silla-Martínez JM, Gabaldón T. trimAl: a tool for automated alignment trimming in large-scale phylogenetic analyses. *Bioinformatics* 2009;25:1972–3. 10.1093/bioinformatics/btp348 19505945 PMC2712344

[80] Minh BQ, Schmidt HA, Chernomor O. et al. IQ-TREE 2: new models and efficient methods for phylogenetic inference in the genomic era. *Mol Biol Evol* 2020;37:1530–4. 10.1093/molbev/msaa015 32011700 PMC7182206

[81] Letunic I, Bork P. Interactive tree of life (iTOL) v5: an online tool for phylogenetic tree display and annotation. *Nucleic Acids Res* 2021;49:W293–6. 10.1093/nar/gkab301 33885785 PMC8265157

[82] Hyatt D, Chen GL, LoCascio PF. et al. Prodigal: prokaryotic gene recognition and translation initiation site identification. *BMC Bioinformatics* 2010;11:119. 10.1186/1471-2105-11-119 20211023 PMC2848648

[83] Eddy SR . Accelerated profile HMM searches. *PLoS Comput Biol* 2011;7:e1002195. 10.1371/journal.pcbi.1002195 22039361 PMC3197634

[84] Johnson LA, Hug LA. Distribution of reactive oxygen species defense mechanisms across domain bacteria. *Free Radic Biol Med* 2019;140:93–102. 10.1016/j.freeradbiomed.2019.03.032 30930298

[85] Jain C, Rodriguez-R LM, Phillippy AM. et al. High throughput ANI analysis of 90K prokaryotic genomes reveals clear species boundaries. *Nat Commun* 2018;9:5114. 10.1038/s41467-018-07641-9 30504855 PMC6269478

[86] Rodriguez-R LM, Konstantinidis KT. The Enveomics Collection: A Toolbox for Specialized Analyses of Microbial Genomes and Metagenomes. *PeerJ Preprints* 2016;4:e1900v1. 10.7287/peerj.preprints.1900v1

[87] ChiPlot . *ChiPlot*. https://www.chiplot.online/.

[88] Eren AM, Kiefl E, Shaiber A. et al. Community-led, integrated, reproducible multi-omics with anvi’o. *Nat Microbiol* 2021;6:3–6. 10.1038/s41564-020-00834-3 33349678 PMC8116326

[89] Eren AM . Vibrio Jascida Pangenome: A Mini Workshop, *Meren Lab*. https://merenlab.org/tutorials/vibrio-jasicida-pangenome/. [Accessed on 25/08/2025].

[90] Harrison KJ, de C-LV, Zallot R. Gene graphics: a genomic neighborhood data visualization web application. *Bioinformatics* 2018;34:1406–8. 10.1093/bioinformatics/btx793 29228171 PMC5905594

[91] Sakai S, Imachi H, Sekiguchi Y. et al. Cultivation of methanogens under low-hydrogen conditions by using the coculture method. *Appl Environ Microbiol* 2009;75:4892–6. 10.1128/AEM.02835-08 19465530 PMC2708418

[92] Sakai S, Conrad R, Liesack W. et al. *Methanocella arvoryzae* sp. nov., a hydrogenotrophic methanogen isolated from rice field soil. *Int J Syst Evol Microbiol* 2010;60:2918–23. 10.1099/ijs.0.020883-0 20097796

[93] Lü Z, Lu Y. *Methanocella conradii* sp. nov., a thermophilic, obligate hydrogenotrophic methanogen, isolated from Chinese rice field soil. *PLoS One* 2012;7:e35279. 10.1371/journal.pone.0035279 22530002 PMC3328440

[94] Mauerhofer LM, Reischl B, Schmider T. et al. Physiology and methane productivity of *Methanobacterium thermaggregans*. *Appl Microbiol Biotechnol* 2018;102:7643–56. 10.1007/s00253-018-9183-2 29959465 PMC6097776

[95] Jasso-Chávez R, Santiago-Martínez MG, Prakash E. et al. Air-adapted *Methanosarcina acetivorans* shows high methane production and develops resistance against oxygen stress. *PLoS One* 2015;10:e0117331. 10.1371/journal.pone.0117331 25706146 PMC4338226

[96] Thauer RK, Kaster AK, Seedorf H. et al. Methanogenic archaea: ecologically relevant differences in energy conservation. *Nat Rev Microbiol* 2008;6:579–91. 10.1038/nrmicro1931 18587410

[97] Aslam S, Lan XR, Zhang BW. et al. Aerobic prokaryotes do not have higher GC contents than anaerobic prokaryotes, but obligate aerobic prokaryotes have. *BMC Evol Biol* 2019;19:35. 10.1186/s12862-019-1365-8 30691392 PMC6350292

[98] Romero H, Pereira E, Naya H. et al. Oxygen and guanine–cytosine profiles in marine environments. *J Mol Evol* 2009;69:203–6. 10.1007/s00239-009-9230-9 19554248 PMC2722718

[99] Varghese NJ, Mukherjee S, Ivanova N. et al. Microbial species delineation using whole genome sequences. *Nucleic Acids Res* 2015;43:6761–71. 10.1093/nar/gkv657 26150420 PMC4538840

[100] Prakash O, Dodsworth JA, Dong X. et al. Proposed minimal standards for description of methanogenic archaea. *Int J Syst Evol Microbiol* 2023;73:005500. 10.1099/ijsem.0.005500 37097839

[101] Riesenfeld CS, Schloss PD, Handelsman J. Metagenomics: genomic analysis of microbial communities. *Annu Rev Genet* 2004;38:525–52. 10.1146/annurev.genet.38.072902.091216 15568985

[102] Jousset A, Bienhold C, Chatzinotas A. et al. Where less may be more: how the rare biosphere pulls ecosystems strings. *ISME J* 2017;11:853–62. 10.1038/ismej.2016.174 28072420 PMC5364357

[103] Lu Y, Conrad R. *in situ* stable isotope probing of methanogenic archaea in the rice rhizosphere. *Science* 2005;309:1088–90. 10.1126/science.1113435 16099988

[104] Keiluweit M, Gee K, Denney A. et al. Anoxic microsites in upland soils dominantly controlled by clay content. *Soil Biol Biochem* 2018;118:42–50. 10.1016/j.soilbio.2017.12.002

[105] Conrad R . Importance of hydrogenotrophic, aceticlastic and methylotrophic methanogenesis for methane production in terrestrial, aquatic and other anoxic environments: a mini review. *Pedosphere* 2020;30:25–39. 10.1016/S1002-0160(18)60052-9

[106] Decho AW, Gutierrez T. Microbial extracellular polymeric substances (EPSs) in ocean systems. *Front Microbiol* 2017;8:922. 10.3389/fmicb.2017.00922 28603518 PMC5445292

[107] Orphan VJ, House CH, Hinrichs KU. et al. Multiple archaeal groups mediate methane oxidation in anoxic cold seep sediments. *Proc Natl Acad Sci USA* 2002;99:7663–8. 10.1073/pnas.072210299 12032340 PMC124316

[108] Tunnacliffe A, Wise MJ. The continuing conundrum of the LEA proteins. *Sci Nat* 2007;94:791–812. 10.1007/s00114-007-0254-y 17479232

[109] Hundertmark M, Hincha DK. LEA (late embryogenesis abundant) proteins and their encoding genes in *Arabidopsis thaliana*. *BMC Genomics* 2008;9:118. 10.1186/1471-2164-9-118 18318901 PMC2292704

[110] Abdul-Aziz M, Sabeem M, Mullath SK. et al. Plant group II LEA proteins: intrinsically disordered structure for multiple functions in response to environmental stresses. *Biomolecules* 2021;11:1662. 10.3390/biom11111662 34827660 PMC8615533

[111] Li XH, Yu CWH, Gomez-Navarro N. et al. Dynamic conformational changes of a tardigrade group-3 late embryogenesis abundant protein modulate membrane biophysical properties. *PNAS Nexus* 2024;3:pgae006. 10.1093/pnasnexus/pgae006 38269070 PMC10808001

[112] Lairson LL, Henrissat B, Davies GJ. et al. Glycosyltransferases: structures, functions, and mechanisms. *Annu Rev Biochem* 2008;77:521–55. 10.1146/annurev.biochem.76.061005.092322 18518825

[113] Whitfield C . Biosynthesis and assembly of capsular polysaccharides in *Escherichia coli*. *Annu Rev Biochem* 2006;75:39–68. 10.1146/annurev.biochem.75.103004.142545 16756484

[114] Sanford RA, Wagner DD, Wu Q. et al. Unexpected nondenitrifier nitrous oxide reductase gene diversity and abundance in soils. *Proc Natl Acad Sci USA* 2012;109:19709–14. 10.1073/pnas.1211238109 23150571 PMC3511753

[115] Jenkins J, Mayans O, Pickersgill R. Structure and evolution of parallel β-helix proteins. *J Struct Biol* 1998;122:236–46. 10.1006/jsbi.1998.3985 9724625

[116] Yoder MD, Keen NT, Jurnak F. New domain motif: the structure of pectate Lyase C, a secreted plant virulence factor. *Science* 1993;260:1503–7. 10.1126/science.8502994 8502994

[117] Boraston AB, Bolam DN, Gilbert HJ. et al. Carbohydrate-binding modules: fine-tuning polysaccharide recognition. *Biochem J* 2004;382:769–81. 10.1042/BJ20040892 15214846 PMC1133952

