# Correction to: Cultivation and genomic characterization of novel methanogens from arid desert biocrust

**DOI:** 10.1093/ismeco/ycag047

**Published:** 2026-06-17

This is a **correction** to:

Weitao Tian, Eva Petrová, Sanae Sakai, Julius Eyiuche Nweze, Anne Daebeler, Roey Angel, Cultivation and genomic characterization of novel methanogens from arid desert biocrust, *ISME Communications*, Volume 6, Issue 1, January 2026, ycag013, https://doi.org/10.1093/ismeco/ycag013

In the originally published version of this manuscript, there are a few minor typographical errors in the text regarding the naming of the new microbes, as registered in SeqCode. These errors are spelling issues and do not affect the scientific content, data interpretation, or conclusions of the article

This error has been corrected.

